# Undergraduate Endodontic Training and Its Relation to Contemporary Practice: Multicenter Cross-Sectional Study in Saudi Arabia

**DOI:** 10.1155/2023/7484570

**Published:** 2023-01-04

**Authors:** Fahda N. Algahtani, Reem M. Barakat, Lujain M. Alqarni, Alanoud F. Alqabbani, Manal F. Alkadi, Rahaf A. Almohareb

**Affiliations:** ^1^Department of Clinical Dental Sciences, College of Dentistry, Princess Nourah Bint Abdulrahman University, P.O. Box 84428, Riyadh 11671, Saudi Arabia; ^2^Dental Intern, College of Dentistry, Princess Nourah Bint Abdulrahman University, P.O. Box 84428, Riyadh 11671, Saudi Arabia

## Abstract

**Materials and Methods:**

An online questionnaire was developed and emailed to all dental schools in Saudi Arabia. This questionnaire was answered by the endodontic undergraduate program director. The data collected were analysed using descriptive statistics.

**Results:**

The response rate was 96.15%, which means twenty-five out of twenty-six dental schools participated in the study. The majority of the academic supervisors was endodontists (92%). The use of magnification and ultrasonic tips was not required by 84% and 76% of the dental schools, respectively. The main endodontic treatment techniques were the step-back technique and gates glidden for cleaning and shaping (76%) and cold lateral compaction for obturation (92%).

**Conclusions:**

The dental students were supervised by endodontic specialists and trained to use traditional endodontic methods. Encouraging dental educators to train students to use modern technology and equipment would probably improve their graduate clinical skills and performance.

## 1. Introduction

General dentists are usually the first to encounter patients in dental pain and provide emergency or complete endodontic care [1, 2]. Root canal treatment (RCT) is a meticulous dental procedure that involves the use of multiple instruments, materials, devices, and radiographs. Therefore, the quality of RCT in dental radiographs is often described as inadequate or substandard [2–5]. The quality of the RCT is one of the main predictors of postoperative peri-radicular healing [6, 7]. Undergraduate students usually feel incompetent in performing one or several steps of RCT [8–11].

The perception of lack of confidence does not necessarily reflect the competence of dental students [12]. However, it may emphasize the complexity of the procedure or the need to increase the extent of undergraduate clinical training. Unfortunately, increasing the time of undergraduate endodontic training may not be permissible since there is a need to achieve competency in several other dental specialties. The European Society of Endodontology emphasized quality over quantity in clinical training. The society also preferred that students be supervised by endodontic specialists. The assumption is that endodontic specialists will improve the standard of supervision in undergraduate training [13]. A lower student-faculty ratio also allows for an individualized learning experience and time for reflection on clinical practice and feedback [14].

Modern endodontic techniques have been developed to overcome the difficulty of RCT [15–17]. Modern endodontics incorporates advanced equipment and materials such as nickel-titanium NiTi files, microscope, and mineral trioxide aggregate (MTA). The introduction of rotary files has improved the operator's efficiency in shaping the root canals. Moreover, it improved the quality of the RCT [15–17]. Furthermore, heat treatment of NiTi files and the use of reciprocation movement have reduced the number of files needed to complete the procedure and improved the shaping quality of RCT [18]. Finally, thermal technology and reciprocating motion significantly increased the cyclic fatigue resistance of rotary files [18–21]. Therefore, the use of heat-treated rotary files and the incorporation of reciprocation motion will increase RCT efficiency, safety, and quality [18–20, 22].

Magnification was also introduced to the field of dentistry to improve clinical precision and posture [23–25]. It becomes necessary in the field of endodontics to improve the predictability and accuracy of the clinical procedure [26–28]. Because it will help the clinician find canals and cracks that are not visible to the naked eye. The dental students found that magnification improved their efficiency and the quality of their performance [29, 30].

Ideally, students need to practice RCT using modern technology and resources that will improve their clinical performance. Although the clinical outcome of the RCT was the same using traditional techniques [31], modern technology allowed for significantly fewer appointment visits, posttreatment interventions, and mishaps [31, 32]. Therefore, progressive advancement elevates the clinical experience for the practitioner and the patient. Since dental patients are usually interested in painless treatment and short dental visits, the American Association of Endodontists has incorporated modern equipment and techniques into the standard of practice for contemporary endodontics [33]. However, international guidelines in endodontic education do not outline materials or equipment that will be needed by dental students [34, 35]. Therefore, the objectives of this study were to explore the current state of endodontic practical training in Saudi dental schools and its relevance to contemporary endodontic practice.

## 2. Materials and Methods

The study was registered, and ethical approval was obtained from the institutional review board of the Princess Nourah Bint Abdulrahman University (IRB: 20-0165).

The questionnaire was developed from a study that was conducted in the United Kingdom about undergraduate endodontic education [36], and it was modified to meet the study objectives. It included ten questions that covered the practical aspect of endodontic practical training, along with three questions about resources available for dental students (Table 1). SurveyMonkey (Momentive Inc., CA, USA) was used to create the online format of the questionnaire.

The names and numbers of all public and private dental schools in Saudi Arabia were obtained from the Ministry of Education website. Further details about dean names and contact information were obtained from the Saudi Dental Education Society. The deans were then contacted to permit study conduction in their institutions and forward the online questionnaire to the undergraduate endodontic program. The data was collected in Excel sheets, and descriptive statistics were used to analyze it.

Endodontic undergraduate training usually commences with preclinical instruction in the third academic year, followed by clinical training in the fourth and final years. There are no national guidelines for undergraduate endodontic education defining a minimum number of required preclinical and clinical training hours [37].

## 3. Results

**Figure 1:**
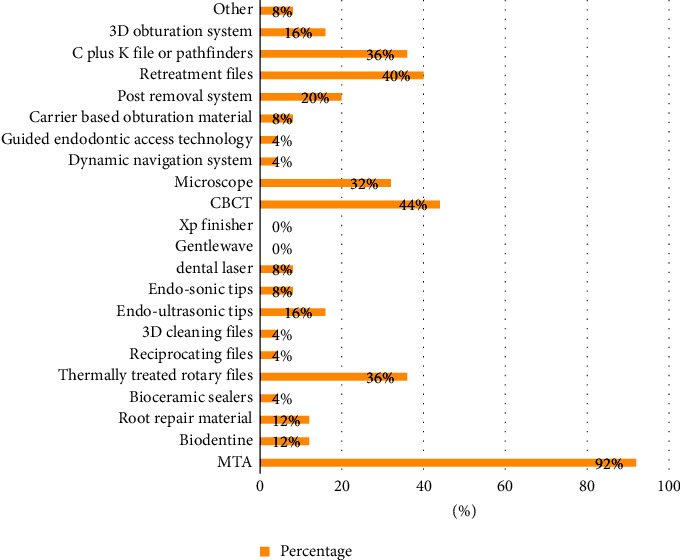
Distribution of the materials and equipment available for dental students' use in dental schools of Saudi Arabia.

Twenty-five out of twenty-six dental schools participated in the study, indicating a response rate of 96.15%. The answers to the survey questions are presented in Table 2. In almost all of these schools (92%), the majority of instructors supervising undergraduate students during their endodontic training were endodontists. The supervisor-student ratio was 1 : 8 or less in preclinical and clinical training. The use of magnification was not applied by 84% of the dental schools (Figure 1). The ultrasonic tips were not used by most schools (76%). Only a few schools use them for removing posts and broken instruments (12%). They are rarely used for access refinement (5%). All dental schools train students to use manual files for RCT (100%), while some dental schools also train students to use rotary files (52%). Students are unlikely to use reciprocation files in their undergraduate training (8%).

The main method of root canal preparation was the step-back technique using K files and gates glidden (76%). Alternatively, students were trained to use the crown-down technique with K files and gates glidden (16%). One school trained the students to use the hybrid technique, using K files and orifice openers as the main shaping techniques. The main irrigants were sodium hypochlorite alone (36%), or sodium hypochlorite and ethylenediaminetetraacetic acid (EDTA) as a final wash (36%). Four dental schools mentioned the use of saline as a final wash after sodium hypochlorite (16%). The main irrigation techniques were passive needle irrigation (68%), and manual agitation (32%).

The main technique of obturation was cold lateral condensation (92%). One dental school trained students to use warm vertical compaction only in preclinical courses, and another school allowed the students to obturate using a single cone technique in addition to the cold lateral condensation technique. Most students were not trained to interpret the CBCT scans (60%). Moreover, 60% of dental schools instruct their students to use calcium hydroxide Ca(OH)_2_ selectively based on the case. The temporization protocol in around half of the dental schools was a spacer and cavit (56%). The alternative protocol is cavit, covered by glass ionomer cement (GIC) or intermediate restorative material (IRM).

Mineral trioxide aggregate (MTA) is available for dental students' use in the majority of dental schools (92%). Some schools provide heat-treated files (36%), retreatment files (40%), microscopes (32%), CBCT (44%), and C + K files (36%), respectively, for dental students. Other advanced equipment and materials, such as reciprocating files, are infrequently made available to undergraduate dental students. Finally, two dental schools mentioned traditional NiTi rotary files as an advanced instrument.

## 4. Discussion

The majority of dental students in the surveyed dental schools are trained by endodontic specialists. The supervision ratio does not exceed one instructor per eight students. Practical training mainly involved traditional techniques in shaping, irrigation, and obturation. The students are not required to use magnification and are taught to use Ca(OH)_2_ selectively, as well as to temporize with a spacer and temporary restoration.

The European Society of Endodontology recommended that dental students' training be ideally supervised by a specialist in endodontics [13]. The present study showed that 92% of dental schools in Saudi Arabia assigned endodontic specialists to supervise their undergraduate dental students. While around 60% to 65% of the United Kingdom and Spain's dental schools provide dental supervisors who are specialists, the supervisor-to-student ratio in Saudi dental schools was approximately similar to the ratio reported in Spain and the United Kingdom [36, 38].

A learning curve is required when adopting rotary NiTi files in root canal instrumentation, since experienced clinicians have fewer endodontic mishaps [39–41]. However, dental clinicians who were trained to use rotary files needed a shorter time to complete the procedure and experienced fewer iatrogenic errors [39–41]. Moreover, manual stainless steel files are less flexible in comparison to NiTi files [42]. Rotary NiTi files conserve the root canal structure and cause less transportation [32]. Therefore, NiTi rotary or reciprocating files can be considered an essential instrument for modern standards of care. The standard of practice for contemporary endodontics in the American Association of Endodontics standard incorporated the use of NiTi files in addition to CBCT, magnification, and endodontic microinstruments [33].

MTA was available for dental students, which is the ideal material for vital pulp therapy and perforation repair [43, 44]. Training students on using the MTA properly will improve treatment outcomes in these situations [43, 44]. Unfortunately, MTA is known to cause discoloration and is associated with handling difficulties [44]. Alternatively, bioceramic materials have been proposed to improve the shortcomings of MTA [45].

In accordance with the present findings, a previous study on dental schools in Spain found that students rarely use magnification and are rarely trained to use ultrasonic tips [38]. Most dental schools in the United Kingdom, on the other hand, teach their students how to use ultrasonic tips [36]. Ultrasonic tips are microinstruments that are frequently used in conjunction with magnification to refine the access, remove the dental blockage, and find the root canals [46]. When endodontists used magnification, the chances of detecting canals such as the second mesiobuccal canal (MB2) in first maxillary molars increased threefold [47, 48]. Therefore, the magnification allowed clinicians to complete the endodontic treatment in a shorter period of time [48]. That said, only 33% of the dental schools in the United Kingdom train students to use loupes and microscopes, and 27% of dental schools train students to use dental loupes only [36]. This is similar to the results found in the present study, where 32% of Saudi dental schools train their students on the use of microscopes. Micro-CT analysis of maxillary molars for the Saudi population revealed that MB2 was detected in 97% of maxillary first molars, and in 70% of these cases, the MB2 canals were not easily located on the pulp floor [49]. Nonetheless, the adoption of magnification loupes in dental training led to better posture, precision, and efficiency for students [29, 30, 50].

Students were mainly encouraged to use Ca(OH)_2_ selectively based on the case and temporize with spacer and cavit. Despite the fact that calcium hydroxide is well known for its antibacterial properties [51–53], the clinical usefulness of this medicament for endodontic procedures is controversial [51–53]. However, no adverse effects were reported when Ca(OH)_2_ was used between dental appointments for a shorter period of time [51–53]. Students should be encouraged to complete the treatment in a single visit since a single-visit endodontic procedure reduces postoperative complications and improves patient compliance [54, 55]. The use of spacer for temporization is also controversial since it may jeopardize the sealing of the root canal system [56, 57]. A recent survey found that the majority of dentists in Saudi Arabia use the same temporization techniques taught in dental schools [58]. Teflon spacers were found to be a better alternative to cotton pellets [59, 60]. However, cavit restoration is only used for less than two weeks and deteriorates easily under masticatory forces [56, 57, 61]. The use of alternative temporization techniques such as cavit covered by glass ionomer can be thoughtfully considered, especially after obturation and for a longer period of temporization [61].

The outcome of RCT in conventional endodontic practice was similar to that in contemporary practice since the objectives of the treatment were the same [31]. Nevertheless, conventional modalities were found to increase treatment time and postoperative interventions [31, 32]. Since evidence suggests that modern technologies and techniques facilitate the efficiency and predictability of RCT, these technologies and techniques will make it easier for graduate dentists to achieve RCT objectives, especially since the quality of root canal treatment has been criticized internationally and nationally [2–5, 31, 32].

Currently, there are no national guidelines for undergraduate endodontic education determining the necessary material and equipment that students should be competent to use; in contrast, the American Association of Endodontists adopted modern techniques and technologies as a standard of practice [33]. Outlining the equipment and materials that have to be available for students will be essential for standard practical training. The development of a national or international framework for undergraduate endodontic education can guide educators in finding consensus that allows them to adopt best practices in education and endodontics [37].

## 5. Conclusions

Dental students were supervised by endodontists, with one endodontic supervisor monitoring up to eight dental students. However, undergraduate dental students were mainly trained to use conventional modalities of treatment. The incorporation of advanced techniques and instruments in endodontic training was recommended.

## Figures and Tables

**Table 1:** The questionnaire.

Practical training	(1) Is there any magnification required for preclinical and clinical endodontic training? (Not required, dental loupes, microscope)
	(2) Are ultrasonic instruments used in pre-clinical and clinical Endodontic training? Check all applicable (not used, access cavity preparation, refinement, dynamic irrigation, post removal, broken instrument removal, other (comment field))
	(3) Select the root canal instruments/systems used in preclinical and clinical endodontic training? Check all applicable (continuous rotation, reciprocation rotation, manual files, other (please specify))
	(4) What is the main method of root canal preparation used? (Step back technique using K files and gates glidden, crown down technique using K files and gates Glidden, a hybrid technique that incorporates K files and rotary orifice opener, rotary files following manufacturer recommendations, reciprocating files following manufacturer recommendations, other (please specify))
	(5) What is the main irrigation routine used in clinical Endodontic training? (Sodium hypochlorite (NaOCL) alone, NaOCL and final wash of EDTA^1^, NaOCL and final wash of EDTA and chlorhexidine, NaOCL and final wash of chlorhexidine, other (please specify))
	(6) What is the main irrigation technique in clinical endodontic training? (Passive needle irrigation, manual agitation, dynamic irrigation using sonic energy, dynamic irrigation using ultrasonic energy, other (please specify))
	(7) What is the main method of root canal filling used in clinical endodontic training? (Single cone, cold lateral compaction, warm vertical compaction, carrier-based obturation, other (please specify))
	(8) Are students trained to interpret CBCT^2^ scans for endodontic purposes? (yes, no)
	(9) Select the regimen for calcium hydroxide (Ca(OH)_2_) medicament placement in undergraduate clinics. (Mandatory in all multivisit treatments, selectively based on etiology, diagnosis, and prognosis, not used)
	(10) What is the protocol for temporizing root canal-treated teeth? A spacer such as cotton pellet and Cavit. Cavit covered with GIC^3^ or IRM^4^. Self or light cured GIC Definitive permanent restoration. Other (please specify)

Resources	(11) What is the qualification of the majority of supervising staff during preclinical and clinical endodontic training? (Specialized in restorative dentistry, specialized in endodontists, general dentistry, advanced general dentistry)
	(12) Please select the supervisors: Student ratio during preclinical/clinical endodontic training. (Preclinical training: 1 : 5 or less, up to 1 : 8, up to 1 : 10, and more than 1 : 10) (clinical training: 1 : 5 or less, up to 1 : 8, up to 1 : 10, and more than 1 : 10)
	(13) Select modern endodontic materials/instruments that are available for use in pre-clinical and clinical endodontic training. Check all applicable. (MTA, Biodentine, root repair material, bioceramic sealers, thermally treated rotary files, reciprocating files, 3D cleaning files, Endo-ultrasonic tips, Endo-sonic tips, dental laser, Gentle Wave, XP finisher, CBCT, microscope, dynamic navigation system, guided endodontic access technology, carrier-based obturation material, postremoval system, retreatment files, C plus *k* file or pathfinders, 3D obturation system, other)

^1^EDTA: ethylenediaminetetraacetic acid, ^2^CBCT: cone beam computed tomography, ^3^GIC: glass ionomer cement, and ^4^IRM: intermediate restorative material.

**Table 2:** The qualification and ratio of dental supervisors in addition to procedural training in Saudi dental schools.

The topic	The answer	No. of dental schools (%)
Qualification of the majority of academic supervisors	Specialized in restorative dentistry	1 (4%)
	Specialized in endodontics	23 (92%)
	Advanced general dentistry	1 (4%)
	General dentistry	0 (0)

Supervisor : students ratio	Preclinical	
	1 : 5 or less	7 (28%)
	Up to 1 : 8	12 (48%)
	Up to 1 : 10	4 (16%)
	More than 1 : 10	2 (8%)
		Total = 25
	Clinical	
	1 : 5 or less	11 (52.2%)
	Up to 1 : 8	7 (30.4%)
	Up to 1 : 10	3 (13%)
	More than 1 : 10	1 (4.4%)
		Total = 22

The use of magnification	Not required	21 (84%)
	Dental loupes	4 (16%)
	Microscope	0 (0)

The use of ultrasonic instruments	Not used	19 (76%)
	Access cavity refinement	5 (5%)
	Dynamic irrigation	0 (0)
	After removal	3 (12%)
	Broken instrument removal	3 (12%)
	Other (comment field)	0 (0)

Root canal instruments/systems used	Continuous rotation	13 (52%)
	Reciprocation rotation	2 (8%)
	Manual files	25 (100%)
	Other (please specify)	0 (0)

The main methods of root canal preparation	Step back technique using K files and gates glidden	19 (76%)
	Crown down technique using K files and gates glidden	1 (4%)
	The hybrid technique that incorporates K files and rotary orifice opener	4 (16%)
	Rotary files	1 (4%)
	Reciprocating files	0 (0)
	Other (please specify))	0 (0)

The main irrigation routine	NaOCl alone	9 (36%)
	NaOCl and a final wash of EDTA	9 (36%)
	NaOCl and a final wash of EDTA and chlorhexidine	3 (12%)
	NaOCl and a final wash of chlorhexidine	0 (0)
	Other (please specify)	4 (16%)

The main irrigation technique	Passive needle irrigation	17 (68%)
	Manual agitation	8 (32%)
	Dynamic irrigation using sonic energy	0 (0)
	Dynamic irrigation using ultrasonic energy	0 (0)
	Other (please specify)	0 (0)

The main method of root canal filling	Single cone	0 (0)
	Cold lateral condensation	23 (92%)
	Warm vertical compaction	0 (0)
	Carrier based obturation	0 (0)
	Other (please specify))	2 (8%)

CBCT scans interpretation	Yes	10 (40%)
	No	15 (60%)

The Ca(OH)2 medicament regimen	Mandatory in all multivisit treatments	10 (40%)
	Selective based on cases	15 (60%)
	Not used	0 (0)

Temporization protocol	A spacer such as cotton pellet and cavit	14 (56%)
	Cavit covered with GIC or IRM	7 (28%)
	Self- or light-cured GIC	0 (0)
	Definitive permanent restoration	0 (0)
	Other (please specify)	4 (16%)

EDTA: ethylenediaminetetraacetic acid; CBCT: cone Beam computed tomography; GIC: glass ionomer cement; IRM: intermediate restorative material.

## Data Availability

The datasets generated and/or analysed during the current study are available as a supplementary file.

## Supplementary Materials

Supplementary Materials S1 includes the whole dataset.
